# Supplementary material for: An insight into the stochastic solitonic features of the Maccari model using the solver technique
Source: PLoS One. 2024 Dec 31;19(12):e0312741. doi: 10.1371/journal.pone.0312741 (PMC11687753; doi:10.1371/journal.pone.0312741)
Supplement: Attachment — Submitted filename: Report.pdf [file pone.0312741.s001.pdf]

A report on the paper entitled "**The structure of stochastic solutions for Maccari model via unified technique**"

The authors used the unified approach to obtain some traveling wave solutions for the coupled Maccari system in Itô sense with multiplicative noise. The solutions were presented in the form of hyperbolic, trigonometric and rational functions. Some two dimensional and three dimensional graphs of some solutions were presented.
This paper contains new ideas and novel analysis. The obtained results seem to be correct and acceptable. The used method is powerful and effective. However, the authors need to consider the following comments.

- In page 3, line 2, please write "in" after the word "found".
- In page 3, line 10, please replace "it's" by "it is".
- In page 3, line 14, please replace the word "describe" by "describes".
- The whole manuscript should be checked for typos and grammatical errors. An overall review is needed for fixing the grammatical and typos errors in the manuscript.
- Punctuation marks should be checked throughout the paper, especially after the equations and at the end of the statements.
- Write the word "Hence," between Eq. (3.5) and Eq. (3.6).
- The introduction should be rearranged in a manner to list the various effects considered in the study and their connection to the existing literature. These are some recent studies to include in the introduction covering various effects used in the present manuscript. I suggest some papers such as:
  https://doi.org/10.32604/cmes.2023.027344
  https://doi.org/10.1016/j.jksus.2022.102087
  https://doi.org/10.3390/fractalfract7030252
  https://doi.org/10.1142/S0218348X23401606
  https://doi.org/10.1080/00036811.2022.2027381

**In conclusion, I think a very minor correction is needed for this paper to be published in the PLOS ONE. I will consider the paper if the above comments are taken into consideration.**
