# Supplementary material for: An insight into the stochastic solitonic features of the Maccari model using the solver technique
Source: PLoS One. 2024 Dec 31;19(12):e0312741. doi: 10.1371/journal.pone.0312741 (PMC11687753; doi:10.1371/journal.pone.0312741)
Supplement: Attachment — Submitted filename: Detailed Responses to Reviewers.pdf [file pone.0312741.s002.pdf]

August 27, 2024

Dear Editor,

First we want to express our deepest gratefulness to the reviewers for their very careful review of our paper and for several valuable comments and suggestions. The following comments are related to our actual version of the enclosed paper named Paper_3_H_Proj1_revised with the changes highlighted in blue colour.

Concerning Reviewer: 1

They investigates the unified approach is used in acquiring some new results to the coupled Maccari system (MS) in Itˆo sense with multiplicative noise. The MS is a nonlinear model used in hydrodynamics, plasma physics, and nonlinear op tics to represent isolated waves in a restricted region. We provide new results with complicated structures to this model, including hyperbolic, trigonometric and ra tional function solutions. We draw the two dimensional (2D) and three dimensional (3D) graphs to some of the study's solutions under appropriately chosen physical parameter values. Random factors can alter the collapse caused by turbulence in the model medium. We noticed that our results may be useful for solving some real-world physical issues by identifying the motion of an isolated wave in a small area. Some points should be discussed and improved.

(1) A comparative study should be included. What is your innovative work? Did you compare your results with others authors results.
Answer: Thank you for pointing this out. We updated Sections one and four with a comparative study. Most typical papers evaluated the suggested model in deterministic situations. In page 2, we also refer to the previous works of the deterministic model. In contrast to our approach, we investigate this model under the stochastic condition, i.e., in the Ito sense of being forced by multiplicative noise. A unified method was employed to discover some innovative random solutions to the MS through the Ito sense.

(2)) The introduction should write according to the following questions: What is already known in the open literature? What is missing (i.e., research gaps)? What needs to be done, why, and how? Clear statements of the novelty of the work should also appear briefly in the Abstract and Conclusions sections. The authors should amend or revise their manuscript based on the following literature. You should cite your paper 2020 to 2023 recent papers: For better presentation of the paper, the authors should cite and discuss re-

cent works on the solitons in this field:
1. Md Mamunur Roshid, Md. Nur Alam, Onur Alp İlhan, Md. Abdur Rahim, Md. Mehedi Hassen Tuhin, M.M. Rahman, Modulation Instability and Comparative Observation of The Effect of Fractional Parameters on New Optical Soliton Solutions of The Paraxial Wave Model, Optical and Quantum Electronics, 56, 1010 (2024). DOI: https://doi.org/10.1007/s11082-024-06921-7 2. Mujahid Iqbal, Md. Nur Alam, Dianchen Lu, Aly R. Seadawy, Nahaa E. Alsubaie, and Salisu Ibrahim, Applications of nonlinear longitudinal wave equation with periodic optical solitons wave structure in magneto electro elastic circular rod, Optical and Quantum Electronics (2024) 56:1031 DOI: https://doi.org/10.1007/s11082-024-06671-6.
Answer: Thank you for pointing this out. We rewritten the introduction according to your questions. We also cited and discussed recent works on the solitons in this field.

Concerning Reviewer: 2
The authors used the unified approach to obtain some traveling wave solutions for the coupled Maccari system in Itˆo sense with multiplicative noise. The solutions were presented in the form of hyperbolic, trigonometric and rational functions. Some two dimensional and three dimensional graphs of some solutions were presented. This paper contains new ideas and novel analysis. The obtained results seem to be correct and acceptable. The used method is powerful and effective. However, the authors need to consider the following comments.

(1) In page 3, line 2, please write ”in” after the word ”found”.
Answer: Thank you for pointing this out. In page 3, line 2, we written ”in” after the word ”found”.

(2) In page 3, line 10, please replace ”it's” by ”it is”.
Answer: Thank you for pointing this out. In page 3, line 10, we replaced ”it's” by ”it is”.

(3) In page 3, line 14, please replace the word ”describe” by ”describes”.
Answer: Thank you for pointing this out. In page 3, line 14, we replaced the word ”describe” by ”describes”.

(4) The whole manuscript should be checked for typos and grammatical errors. An overall review is needed for fixing the grammatical and typos errors in the manuscript.
Answer: Thank you for pointing this out. We carefully checked the whole paper for typos and grammatical errors. We also fixed the grammatical and typos errors in the manuscript.

(5) Punctuation marks should be checked throughout the paper, especially after

the equations and at the end of the statements.
Answer: Thank you for pointing this out. We carefully checked the punctuation throughout the paper.

(6) Write the word ”Hence,” between Eq. (3.5) and Eq. (3.6).
Answer: Thank you for pointing this out. We write the word ”Hence,” between Eq. (3.5) and Eq. (3.6).

(7) The introduction should be rearranged in a manner to list the various effects considered in the study and their connection to the existing literature. These are some recent studies to include in the introduction covering various effects used in the present manuscript.
Answer: Thank you for pointing this out. We rearranged the introduction in a manner to list the various effects considered in the study and their connection to the existing literature. We enriched the reference section with very relevant papers.

Concerning Reviewer: 3

(1) The title of this research manuscript should read ”An insight into the stochastic solitonic features of the Maccari model using the solver technique instead of ”The structure of stochastic solutions for Maccari model via unified technique”.
Answer: Thank you for pointing this out. We replaced the title of this research manuscript as ”An insight into the stochastic solitonic features of the Maccari model using the solver technique” instead of ” The structure of stochastic solutions for Maccari model via unified technique”.

(2) What are the key features of the proposed technique.
Answer: Thank you for pointing this out. In page 3, below Eq. (1.2), we mentioned the key features of the proposed technique.

(3) The significance of the obtained solutions should be highlighted.
Answer: Thank you for pointing this out. In Section 4, we highlighted the significance of the obtained solutions.

(4) In the introduction section, authors should present some properties of Browning motion process.
Answer: Thank you for pointing this out. In page 3, we presented some properties of Browning motion process.

(5) The authors must clearly indicate what is the difference between the present work history and other research work.
Answer: Thank you for pointing this out. In Sections 1 and 4, we clearly indicated what is the difference between the present work history and

other research work. Indeed, most standard papers evaluated the suggested model in deterministic case. In page 2, we also refer to the previous works of the deterministic model. In contrast to our approach, we investigate this model under the stochastic condition, i.e., in the Ito sense of being forced by multiplicative noise. A unified method was employed to discover some innovative random solutions to the MS through the Ito sense.

(6) Revise the whole manuscript for punctuation issues, such as end of equations (3.2).
Answer: Thank you for pointing this out. We revised the whole manuscript for punctuation issues.

(7) The authors should state in the paper what kind of software package has been used to obtain the required results.
Answer: Thank you for pointing this out. In section 4, we stated what kind of software package has been used to obtain the required results. Namely, we provide some 2D and 3D graphs for some chosen solutions of the proposed model for appropriate parametric choices using Matlab version 18.

(8) The entire manuscript should be totally double-checked for typographical errors, text inconsistencies.
Answer: Thank you for pointing this out. We carefully checked the entire manuscript for typographical errors, text inconsistencies.
The entire manuscript should be totally double-checked for typographical errors, text inconsistencies

We are looking forward for your decision.

[Please do not hesitate to contact us if further revisions are needed].

With best regards

Sincerely
